# Supplementary material for: Thoughts on the potential to compensate a hearing loss in noise
Source: F1000Res. 2021 Apr 22;10:311. [Version 1] doi: 10.12688/f1000research.51784.1 (PMC8524304; doi:10.12688/f1000research.51784.1)
Supplement: Peer review history

# Peer review: Reviewer response for version 1

**DOI:** 10.5256/f1000research.54989.r96389

**Published:** 2021-10-18

**Authors:** Arne Leijon

**Affiliations:** 1 Retired from School of Computer Science and Communication, KTH Royal Institute of Technology, Stockholm, Sweden

**Main Comments**

 This is a good paper. I agree with the author that it contributes to the scientific topic that may rightly be called “ *theoretical Audology*”:

 The paper summarizes several earlier studies indicating that the specific difficulty of people with impaired hearing to understand speech in noise is caused by a reduced information-transmission capacity of the peripheral auditory system ("class D" loss). These earlier results also suggest that the information loss has similar effects as an artificially induced spectro-temporal *level uncertainty* in the front-end processor of a simulation system for auditory discrimination (FADE), which uses an automatic speech recognition system to quantify the effects on speech recognition.

 Considering the hypothesis that the level uncertainty in the computational auditory model might correspond to some physiological processes in the auditory system of real people, the author has designed a signal-processing system (PLATT) that can be used in real hearing aids to compensate for the level uncertainty, by enhancing spectral modulation patterns that are most important to convey phonetic contrasts in real speech.

 The paper clearly explains the main features of the PLATT system, and presents simulation results showing that about half of the simulated loss caused  by the level uncertainty could be compensated by hearing-aid spectral expansion. Of course, the results from model simulations in a computer might be seen as just an advanced form of mathematical speculation, and the author wisely emphasises that these simulation results cannot prove that PLATT is beneficial for real listeners.

 Instead, the paper uses the simulation results as a starting point to discuss how an empirical speech recognition experiment should be designed to be able to test the two hypotheses, (1) that something like the modelled level uncertainty causes the speech-recognition loss in real listeners, and (2) that spectral modulation enhancement in a hearing aid can (at least partly) compensate for this loss.

 The paper shows that it is far from trivial to design such an experiment with real listeners. The experiment should separate the specific effects of modulation expansion from the effects of linear (or slow-compression) amplification, because both types of processing can have effects on speech recognition, loudness, and subjective quality of the processed speech in noise. Another serious difficulty is the test-retest uncertainty of all speech-recognition test methods.

 I think all of this discussion is scientifically valuable and should be approved for indexing. I have only a few suggestions to make the presentation easier to read and understand. (I have not studied the code for the FADE and the  PLATT system, but I think I have understood the approach and I appreciate that the code has been made publicly available.)

**Suggestions for Clarity**:

**Temporal modulation patterns**: The second point in the Conclusions states that hearing aids “… only allow for a manipulation of mainly spectral modulation patterns.”

 This formulation seems a little too strong: All compression systems have some effects on the temporal “modulation patterns” of the input signal. For example, slow automatic gain control (AGC) (with long attack time and long release time) tends to compress only the very slow temporal modulations while leaving faster modulations nearly unchanged. People have also studied AGC systems controlled by several signal features in parallel, with different time constants, with more complex effects on the temporal modulation patterns. The proposed PLATT system also “manipulates” the temporal “modulation patterns”, although it was not specifically designed for that purpose.

**Figure Captions and Table Header**:

 I guess I am not the only reader tempted to look at the figures before reading the detailed explanations in the surrounding text. I understand the figure caption can not include a long complicated explanation, but, to facilitate understanding, the figure captions should at least define all graphical elements seen in the figure. No problem if the caption gets long.

 Examples:

**Figure 9:** This figure is complicated but very important for readers to understand, as it is central for the explanation of PLATT. The caption should clearly state that the labels 1,2,3,4 refer to the *differences* between the displayed curves, because text labels in a graph most often refer to the plotted curves, not to the *distance* between curves. Perhaps this could be even more clearly shown graphically by a vertical line indicating the difference, connected to the arrow line to the label.

 Also, at first sight, I was confused by the use of grayscale: The lightest gray curve in the GAIN plot is *not* the difference between the correspondingly light-gray curves in the OUTPUT and the INPUT plots, which would be the default interpretation. Instead, a gray GAIN curve is the difference between the gray OUTPUT (partial) spectrum and the solid black curve for the total INPUT spectrum. This should be explained in the caption. I spent some time searching the text before I found this explanation.

**Figure 10**: The caption text seems to suggest that there are three spectra with only pure tones, and another three spectra with added white noise, but there is only one dotted curve, showing the spectrum of the pure tones with peaks about 60 dB SPL with added white noise. (In this figure, the gray GAIN curves are actually the difference between similarly gray OUTPUT and INPUT spectra, contrary to the use of gray-scale in Fig 9.)

**Figures 12-18**: The second and fourth plot titles state “difference in SRT”, but the plot actually shows a positive “difference in SRT”, i.e., an increased SRT as a negative number, because it indicates a decrease in performance. This is of course easy to understand, but should still be mentioned in the first caption. Also, the meaning of legend text strings, like “P-8000-1 none” must be explained in the caption. At first sight of Fig 12, I could not guess the intended meaning of “none”.

**Table 1**: The column with heading “NH” should be explained more clearly in the table header. The numerical values in this column do actually *not* indicate “the normal-hearing listener profile” as stated. The numbers show the *difference* between results for the NH and the unaided impaired profile on the same row in the first column. I needed to read the explanation on the following page before I understood that the numbers in the “NH” column are meant to be interpreted as ideal measures to which the benefit measures shown in the “PLATT-x” columns might be compared.

Is the work clearly and accurately presented and does it cite the current literature?

Yes

If applicable, is the statistical analysis and its interpretation appropriate?

Yes

Are all the source data underlying the results available to ensure full reproducibility?

Yes

Is the study design appropriate and is the work technically sound?

Yes

Are the conclusions drawn adequately supported by the results?

Yes

Are sufficient details of methods and analysis provided to allow replication by others?

Yes

Reviewer Expertise:

Probabilistic pattern recognition, model-based signal processing, hearing technology, psycho-acoustics.

I confirm that I have read this submission and believe that I have an appropriate level of expertise to confirm that it is of an acceptable scientific standard.

# Peer review: Reviewer response for version 1

**DOI:** 10.5256/f1000research.54989.r83763

**Published:** 2021-05-17

**Authors:** Jon Barker

**Affiliations:** 1 Department of Computer Science, University of Sheffield, Sheffield, UK

The paper considers hearing impairment as a sum of loss due to attenuation and loss due to distortion, as proposed by Plomp (1978). It considers, in particular, the contribution of the distortion component (modelled as internal noise) and asks how much the deficit caused by this component might be restored by well-designed dynamic range manipulation. A patented dynamic range manipulation scheme (PLATT) is presented which works by decomposing spectral modulations into different frequency bands and applying a compression scheme that treats each band separately. The benefit of PLATT for ameliorating distortion loss is then tested by measuring the predicted SRT-50 improvements for speech in different noise conditions and listeners with different hearing profiles (where distortion loss if modelled by adding level uncertainty to the spectro-temporal signal representation). These experiments have used conditions that isolate the effect of distortion loss (i.e., comparing conditions with identical attenuation loss).  The FADE (ASR-based) model is used to predict listener behaviour.

 The author is clear to point out that the purpose of the paper is not to make specific claims for the benefit of PLATT in real listening conditions, but instead, to make specific and testable predictions about the outcomes of (yet-to-be-performed) listening experiments. In particular, the experiments will test the hypothesis that the distortion component of hearing loss can be modelled by level-uncertainty. The paper concludes by discussing the design of the listening studies and by presenting thoughts on the future development and evaluation of PLATT in real-world listening conditions where both distortion and attenuation need to be considered.

 I greatly enjoyed reading this well-written, detailed and thought-provoking paper. I have no major criticisms but have listed a few points for clarification below and a number of specific typos that can be fixed.

**For clarification**

- On page 19, the paper says "the curves show some variability which is due to the stochastic nature of the simulation process." I was not quite sure how to interpret this. Is this stochasticity in the FADE model, e.g. in the ASR training and testing? If so could the variability in the result be reduced by averaging over a greater number of simulated runs. If not would it be useful to add error bars to the Plomp plots.
- On page 26, the hypothesis and outcomes of the future listening experiments are very clearly stated, and it is anticipated that the PLATT expansion may improve SRTs by less than predicted. It is said that this will be taken to mean that increased level uncertainty causes only a part of the class D hearing loss. However, could there be other reasons why listeners would find it hard to process the PLATT signals. For example, they might successfully reduce uncertainty but produce signals that do not match their expectations of speech signals, i.e., in FADE the ASR system is trained extensively on the processed signal, but in the listening experiments, listeners have their own internal speech models. Would listeners need extensive listening to adapt to the signals?
- On page 29 it is noted that FADE is a poor predictor of SRTs for speech in the presence of speech interferers. I think it would be interesting to say a little about why this might be the case. Is it that it does not explicitly model informational masking effects, or that it does not model source separation? And if so are these factors that could be at play in the processing of the speech plus ICRAS-250 noise? And could these factors interact with the PLATT processing? e.g., cues for source separation being compromised by the non-linear PLATT processing.  

**Specific points and typographical errors,**

 Page 3,"This limit is still way above" -> "This limit is still far above" (more formal and more easily understood)

 Page 8, end of first paragraph, "be performed human listeners" -> "be performed by human listeners"

 Page 10, "fewer frequency channels reduce" -> "using fewer frequency channels reduces"

 Page 11, "raises the question which representation" -> "raises the question of which representation"

 Page 11, Figure 5, please remake with larger text

 Page 12, after eq (1), "and f_b the sampling frequency" -> "and f_s the sampling frequency" (or change f_s to f_b in the equation)

 Page 13, Spectro-temporal signal representation: the envelope estimate would be clearer if presented with an equation. 

 Page 16, after 1760 at end of page add, (2 x 11 x 16 x 5) just to be clear about how the total number of conditions arises.

 Page 18, line 2, "as one might have expect." -> "as one might have expected."

 Page 18, start of final paragraph, "present effect of" -> "present the effect of"

 Page 19, paragraph 2, "due to PLATT in was" -> "due to PLATT was"

 Page 25, final paragraph, "with an specific" -> "with a specific"

Is the work clearly and accurately presented and does it cite the current literature?

Yes

If applicable, is the statistical analysis and its interpretation appropriate?

Not applicable

Are all the source data underlying the results available to ensure full reproducibility?

Yes

Is the study design appropriate and is the work technically sound?

Yes

Are the conclusions drawn adequately supported by the results?

Yes

Are sufficient details of methods and analysis provided to allow replication by others?

Yes

Reviewer Expertise:

Automatic speech recognition, machine listening

I confirm that I have read this submission and believe that I have an appropriate level of expertise to confirm that it is of an acceptable scientific standard.
